# Thyroid Autoimmunity and Function after Treatment with Biological Antirheumatic Agents in Rheumatoid Arthritis

**DOI:** 10.3389/fendo.2017.00179

**Published:** 2017-07-31

**Authors:** Sofie Bliddal, Stina Willemoes Borresen, Ulla Feldt-Rasmussen

**Affiliations:** ^1^Department of Medical Endocrinology, Copenhagen University Hospital, Rigshospitalet, Copenhagen, Denmark

**Keywords:** autoimmune thyroiditis, rheumatoid arthritis, biological antirheumatic agents, Hashimoto’s thyroiditis, Graves’ disease, thyroperoxidase antibody, thyroglobulin antibody, tumor necrosis factor-alpha inhibitors

## Abstract

With the increased pro-inflammatory response in both rheumatoid arthritis and thyroid autoimmune diseases, treatment with biological antirheumatic agents (BAAs) of the former may affect the course of the latter. In hepatitis C and cancer patients, treatment with biological agents substantially increases the risk of developing thyroid autoimmunity. As the use of BAAs in the treatment of rheumatoid arthritis is increasing, this review aimed to investigate if such use affected thyroid status in rheumatoid arthritis patients. We conducted a systematic literature search and included six studies with a total of 311 patients as well as three case reports. The patients were treated with tumor necrosis factor-α inhibitors (infliximab, etanercept, or adalimumab) or the monoclonal CD20-antibody rituximab. There was a non-significant trend of slight improvement of both thyroid function and autoantibody status: a reduction of thyroid peroxidase and thyroglobulin antibody concentrations, and a reduction of thyrotropin levels in hypothyroid patients. Despite the small number of studies, they presented compliant data. The BAAs used in rheumatoid arthritis thus did not seem to negatively affect thyroid status in patients with rheumatoid arthritis and can be considered safe with regard to thyroid autoimmunity. However, the well-established association between rheumatic diseases and thyroid autoimmunity necessitates continued monitoring of thyroid function in patients with rheumatoid arthritis. Each new BAA should be scrutinized for its effect on thyroid as well as other autoimmune diseases in order to establish concise recommendations for patient follow-up for each agent and each disease.

## Introduction

Autoimmune thyroid diseases are the most common autoimmune diseases and are often associated with the presence of other organ-specific or non-organ-specific autoimmune diseases (1). The coexistence of thyroid autoimmunity and rheumatoid arthritis has been acknowledged for over a century (2). Population studies have confirmed an increased prevalence of autoimmune thyroid disease in patients with rheumatoid arthritis and conversely, an increased prevalence of rheumatoid arthritis in patients with autoimmune thyroid disease (3, 4). Boelaert et al. (5) asked 3,286 thyroid patients to report other autoimmune diagnoses among themselves and their relatives. Rheumatoid arthritis was the most prevalent coexisting autoimmune disease occurring in 3.15% of patients with Graves’ disease and 4.24% of patients with Hashimoto’s thyroiditis (5). Similarly, Fallahi et al. (6) found a 2.4% prevalence of rheumatoid arthritis in 3,069 patients with verified autoimmune thyroiditis, which was significantly higher than in patients with multinodular goiter and in thyroid-healthy age- and sex-matched controls (*p* < 0.0001). In a recent meta-analysis (7), patients with rheumatoid arthritis had a three times higher risk of having thyroid autoantibodies than healthy controls [thyroglobulin autoantibody (TgAb): OR 3.17 (2.24–4.49) and thyroglobulin autoantibody (TPOAb): OR 2.33 (1.24–4.39)].

Although an association between thyroid autoimmunity and rheumatoid arthritis has been demonstrated, the causality is not yet established. However, there is increasing awareness of a possible common pathogenesis behind autoimmune diseases potentially caused by an underlying immunological breach resulting in disruption of self-tolerance (8). It is generally believed that both autoimmune thyroid disease and rheumatoid arthritis occur as a result of multiple factors (genetic susceptibility, endogenous, and environmental) (1, 9, 10). A malfunctioning T and B cell regulation causing reactions against autoantigens is involved in both conditions with antibody production in rheumatoid arthritis (rheumatoid factor and anti-cyclic citrullinated peptide) and thyroid autoimmunity (thyroid peroxidase, thyroglobulin, and thyroid stimulating hormone receptor antibodies) (1). Such T and B cell regulation is highly complex, but is intertwined with expression of various cytokines. Both cytokine production and B cell function are among the targets of newer biological antirheumatic agents (BAAs), which are increasingly used in the treatment of rheumatoid arthritis (11, 12). Current guidelines generally recommend treatment with BAAs when there is either insufficient response to treatment with the conventional disease modifying antirheumatic drugs or in the presence of unfavorable prognostic markers (autoantibodies, high disease activity, early erosions, and failure of two conventional disease modifying antirheumatic drugs) (11, 12). Although an increasing amount of antirheumatic agents exist, the most commonly used in the treatment of rheumatoid arthritis is the group of inhibitors of tumor necrosis factor-α (TNF-α). This pro-inflammatory cytokine plays a vital role in the immunological activation related to the autoimmune inflammatory patterns in rheumatoid arthritis. TNF-α expression has also been shown to be increased in patients with autoimmune thyroid disease, and therefore, treatment with TNF-α inhibitors could possibly effect thyroid autoimmune status as well (13). In previous studies of older immunomodulatory agents (e.g., interferon-α) in patients with multiple sclerosis and hepatitis C, it has long been known that thyroid autoimmunity develop in more than one-third of such patients (14–16). A mutual affection of the immune system in rheumatoid arthritis and thyroid autoimmunity makes the use of BAAs relevant also within the field of thyroid autoimmunity. The present review investigates the association between biological antirheumatic treatment of rheumatoid arthritis and affection of thyroid autoimmunity.

## Methods

In March 2017, a Medline literature search was performed using key terms of “rheumatoid arthritis,” “thyroid,” and alternately generic and commercial names of known BAAs. Identified studies were initially screened by title and abstract, and full text retrieved for further scrutiny. Further, the reference lists of included studies were checked. Only original studies evaluating thyroid autoimmunity during treatment with BAAs for rheumatoid arthritis were eligible for inclusion. Case reports were included and presented separately. All relevant data were carefully extracted from each included paper and txabulated independently by two authors (Sofie Bliddal and Stina Willemoes Borresen). Extracted data included: first author, publication year, number of patients, the studied BAA, patients’ other medication, follow-up, method and cut-off used for detecting thyroid antibodies, known autoimmune thyroid disease, and thyroid status including autoimmunity before and after treatment with the BAA. Any disagreements were resolved by consensus. In some studies, the proportion of patients with positive thyroid antibodies was not reported directly, but could be calculated from study data.

## Results

### Study Characteristics

A total of 14 relevant articles were identified. Upon scrutiny, five articles failed to comply with the inclusion criteria or had insufficient outcome reports of thyroid status, leaving six included articles (17–22), in a total 311 patients, and three were case reports (23–25). The studies included five prospective cohort studies of previous BAA-naïve patients with rheumatoid arthritis (17–20, 22) and one cross-sectional study of patients with rheumatoid arthritis who had received treatment with BAAs (Table 1) (21). The TNF-α inhibitors adalimumab (ADM), infliximab (INX), and etanercept (ETC) were used in three (17, 21, 22), four (18–21), and two (18, 21) studies, respectively. The monoclonal CD20 antibody rituximab (RIX) was used in two studies (20, 21). One of these was a cross-sectional study (21) and the other did not stratify results from patients treated with RIX from results of patients treated with the TNF-α inhibitor INX (20). One study (20) excluded patients with previously known thyroid disease, three studies (17, 21, 22) and two studies (18, 19) did not report whether they excluded these patients. All six studies reported the number of TPOAb-positive patients, and three studies (17, 20, 21) reported TgAb positivity. Additionally, two studies (20, 22) reported the TPOAb levels and one study (20) reported the TgAb levels. Two studies (20, 22) reported mean TSH and free thyroxine (FT4) levels.

**Table 1:** Studies of thyroid status in rheumatoid arthritis patients treated with BAAs.

Reference	Study group	BAA	Dosage	Other treatment	Antibody assay	Antibody cut-off	Baseline		Follow-up time	Outcome	
									
							Antibody positivity	Thyroid function		Antibody positivity	Thyroid function
Atzeni et al. (17)	20 RA pts (17 women)	ADM	40 mg/2 weeks	MTX: 20/20 (100%) GC: 13/20 (65%) NSAID: 14/20 (70%)	ICMA (immulite 2000, DPC)	TPOAb: 35 IU/mL TgAb: 40 IU/mL	TPOAb: 6/20 (30%) TgAb: 8/20 (40%)	HT: 3/20 (15%)	6 months	TPOAb: 5/20 (25%) TgAb: 8/20 (40%)	NA

Caramaschi et al. (18)	54 RA pts (46 women)	INX *n* = 43 ETC *n* = 11	INX 3 mg/kg onweek 0, 2, 6 and every 8 weeks ETC 2 × 25 mg/week	MTX: 52/54 (96%) Azathioprine: 2/54 (4%)	ELISA	TPOAb: 35 IU/mL TgAb: 40 IU/mL	TPOAb: 6/54 (11%) TgAb: 7/54 (13%) +4 Ab positive pts^a^	NA	12 months	TPOAb: 6/54 (11%), TgAb: 7/54 (13%) (12 INX, 1 ETC) Shift Ab-neg to Ab-pos: 6 pts Shift Ab-pos to Ab-neg: 4 pts (INX)^a^	All euthyroid

Elkayam et al. (19)	26 RA pts (17 women)	INX	3 mg/kg on week 0, 2, 6, and every 8 weeks	MTX 26/26 (100%)	ELISA (Zeus Scientific)	TPOAb: 25 IU/mL	TPOAb: 0/26	NA	14 weeks	TPOAb: 0/26	NA

Kaklamanos et al. (20)	36 rheumatic pts^b^ (28 women)	INX *n* = 18 (14 RA pts) RIX *n* = 18 (12 RA pts)	INX 200, 350, or 500 mg RIX 2 × 1,000 mg/2 weeks or 4 × 500 mg/week	MTX: 13/36 (36%) Prednisolone: 16/36 (44%) Leflunomide: 3/36 (8%) HCQ: 2/36 (6%)	CMIA (Architect i2000, Abbott)	TPOAb: 5.61 IU/mL TgAb: 4.11 IU/mL	TPOAb: 4/36 (11%) TgAb: 6/36 (17%)\ TPOAb level (mean ± SD): 36.8 ± 44.9 TgAb level (mean ± SD): 10.6 ± 7.1	TSH (mean ± SD): 1.7 ± 1.2 mU/L FT4 (mean ± SD): 15.3 ± 3.7 pmol/L	3 years	TPOAb: 4/36 (11%) TgAb: 5/36 (14%) TPOAb level (mean ± SD): 20.2 ± 16.7 TgAb level (mean ± SD): 9.3 ± 5.5	TSH (mean ± SD): 1.8 ± 1.2 mU/L FT4 (mean ± SD): 15.4 ± 3.6 pmol/L

Koszarny et al. (21)	37 RA pts with a history of BAA treatment^c^	INX, ETC, ADM, or RIX^d^	NA	MTX, other DMARDs, prednisolone^d^	ELISA (Euroimmun)	TPOAb: 50 IU/mL TgAb: 100 IU/mL TRAb: 2 IU/mL	NA	NA	NA (cross-sectional)	TPOAb: 4/37 (11%) TgAb: 2/37 (5%)	NA

Raterman et al. (22)	138 RA pts with known thyroid status (106 women)	ADM	NA	MTX 108/138 (78%) Prednisolone 47/138 (34%) No. DMARDs used: 2–6	ELISA (Cobas^®^ analyzer)	TPOAb: 34 IU/mL	TPOAb: 21/138 (15%) TPOAb level (median): 267 IU/mL	Mean TSH: 1.5 mU/L	6 months	TPOAb: 20/138 (15%) TPOAb level 201 IU/mL*	Mean TSH: 1.3 mU/L*

	Hypothyroid pts						TPOAb: 11/18 (61%)	18/138 (13%)	6 months	TPOAb: 12/18 (67%)	16/138 (12%)
							TPOAb level (median): 325 IU/mL	Mean TSH: 4.1 mU/L		TPOAb level (median) 282 IU/mL	Mean TSH: 3.5 mU/L
	Euthyroid pts						TPOAb: 9/113 (8%)	113/138 (82%)	6 months	TPOAb: 7/113 (6%)	7/138 (5%)
							TPOAb level (median): 114 IU/mL	Mean TSH: 1.4 mU/L		TPOAb level (median) 108 IU/mL	Mean TSH: 1.3 mU/L
	Hyperthyroid pts						TPOAb: 1/7 (14%)	7/138 (5%)	6 months	TPOAb: 1/7 (14%)	7/138 (5%)
							TPOAb level: 73 IU/mL	Mean TSH: 0.5 mU/L		TPOAb level 49 IU/mL	Mean TSH: 0.5 mU/L

*^a^Not reported whether TPOAb and/or TgAb*.

*^b^Twenty-six patients with RA, six patients with SLE, four patients with sero-negative arthritis. The study further included three different control groups*.

*^c^The number of female patients not reported. The study also included 38 patients BAA-naïve RA patients*.

*^d^The number of patients treated with each drug not reported*.

*p < 0.05 compared to baseline data.

*ADM, adalimumab; BAAs, biological antirheumatic agents; CMIA, Chemiluminescent microparticle immunoassay; ETC, etanercept; HCQ, Hydroxychloroquine; FT4, free thyroxine; HT, Hashimoto’s thyroiditis; ICMA, immunochemiluminescence assay; INX, infliximab; MTX, methotrexate; NA, data not available; pts, patients; RA, rheumatoid arthritis; RIX, rituximab; TgAbs, thyroglobulin autoantibodies; TPOAbs, thyroid peroxidase autoantibodies; TRAbs, thyrotropin receptor autoantibodies*.

### Thyroid Autoimmunity

**Figure 1:**
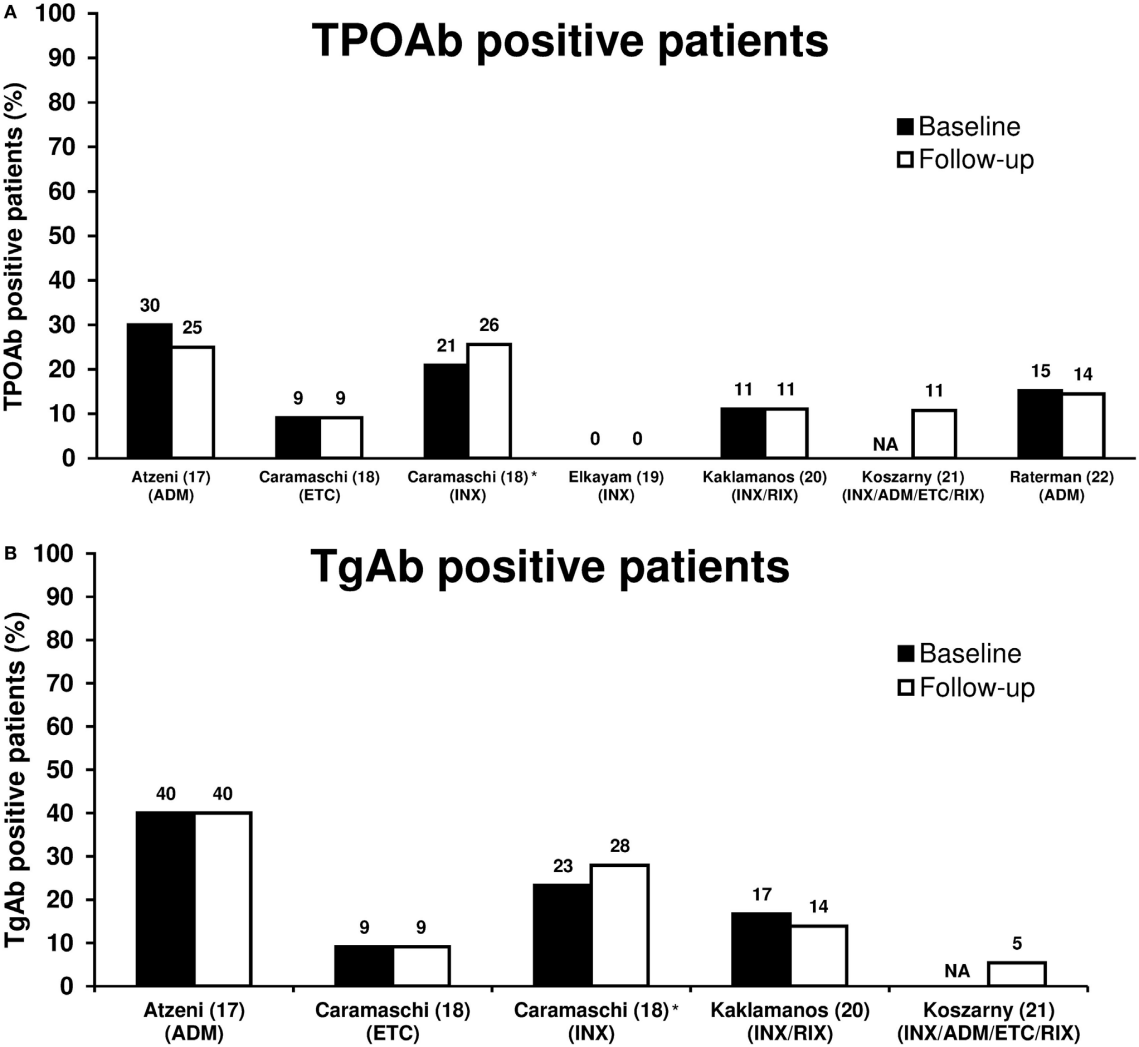
Proportion of TPOAb-positive **(A)** and TgAb-positive **(B)** patients at baseline and follow-up in patients with rheumatoid arthritis treated with biological antirheumatic agents (BAAs). Studies are presented by first author name (reference number) (BAA). *In four patients at baseline and six patients at follow-up, no distinction was made between TPOAb-positivity and TgAb-positivity, and thus the reported thyroid autoantibody prevalence was included in both Figures 1A,B. Abbreviations: ADM, adalimumab; ETC, etanercept; INX, infliximab; RIX, rituximab; NA, not available. TgAb, thyroglobulin autoantibody; TPOAb, thyroid peroxidase autoantibody.

Biological antirheumatic treatment did not seem to affect the number of TPOAb-positive patients, which varied from 0 to 30% of patients at baseline and 0 to 25% at follow-up (Figure 1A; Table 1). Two studies reported a single patient turning TPOAb-positive after treatment with ADM (17, 22), and the number of TPOAb-positive patients was unchanged in two studies of INX (19) or INX/RIX (20), respectively. Likewise, only one TgAb-positive patient turned antibody-negative and none in a study of ADM (17) (Figure 1B). In the study of Caramaschi et al. (18), six patients treated with INX shifted from negative to positive thyroid antibodies and four patients shifted from positive to negative antibodies (no distinction was made between TPOAb and TgAb positivity). In a cross-sectional study (21), TPOAb-positivity was found in 4/37 (11%) patients with a history of biological antirheumatic treatment, which was similar to the 3/38 (8%) of patients who were BAA-naïve. The corresponding numbers of TgAb-positive patients were 2/37 (5%) and 4/38 (11%) for patients who had or had not received biological antirheumatic treatment, respectively.

Decreased or unchanged thyroid antibody levels were reported in two studies. In Raterman et al. (22), the mean TPOAb concentration decreased from 267 to 201 IU/mL (*p* = 0.048) in TPOAb-positive patients after 6 months of treatment with ADM. In Kaklamanos et al. (20), mean TPOAb concentration decreased insignificantly from 36.8 to 20.2 IU/mL in TPOAb-positive patients after 24–40 months treatment with INX or RIX. Likewise, mean TgAb concentration was unaffected in TgAb-positive patients (mean TgAb level 10.6–9.3 IU/mL) (20).

### Thyroid Function

Two studies investigated changes in thyroid function before and after biological antirheumatic treatment (20, 22). In Raterman et al. (22), treatment with ADM led to a decrease in mean TSH level from 1.5 to 1.3 mU/L (*p* = 0.0014) in the total group, whereas FT4 levels did not change. The decrease in TSH was larger in (previous) hypothyroid patients compared with euthyroid patients, but TSH also decreased in 8/10 of the hypothyroid patients who were not treated with l-thyroxine. Two of these patients became euthyroid after 6 months treatment with ADM (22). In Kaklamanos et al. (20), mean TSH changed from 1.7 to 1.8 IU/L, and FT4 changed from 15.3 to 15.4 pmol/L, yet both not significantly.

### Cases

Our search revealed three case reports of autoimmune thyroiditis in patients with rheumatoid arthritis treated with a BAA. Two cases (23, 24) reported hyperthyroidism in patients treated with anti-TNFα. The first case (23) reported transient hyperthyroidism, lasting 1 month, in a patient after 6 months of treatment with ETC (TPOAb and TgAb negative, TRAb not measured). In a second case report (24), a 70-year-old women developed Graves’ disease after 8 years of treatment with ADM. Interestingly, one case reported improvement of previously known autoimmune hypothyroidism in a patient after treatment with RIX (25). TPOAbs declined to undetectable levels after 6 months of treatment with RIX, and in an unchanged l-thyroxine dose the patient became clinically hyperthyroid with a TSH decline from 1.18 to 0.10 mU/L (25).

## Discussion

The present review showed that the BAAs used to treat rheumatoid arthritis did not seem to induce or worsen autoimmune thyroid disease. On the contrary, there was a tendency toward a positive effect; a reduction of TPOAb and TgAb concentrations and a reduction of TSH levels in hypothyroid patients. Despite the small number of included studies with diverse immunomodulatory agents (mainly TNF-α inhibitors, a few of RIX), the studies presented compliant data.

Due to its multiple immunological mechanisms, TNF-α has been previously investigated in thyroid patients. Both hypo- and hyperthyroid patients had significantly higher levels of TNF-α compared to controls, and in hyperthyroid patients successful treatment led to normalization of TNF-α levels (13). However, as demonstrated in the cases reported by van Lieshout (24) and Allanore (23), hyperthyroidism was diagnosed after initiation of anti-TNF-α therapy. It is difficult to assess whether this could be attributed to the treatment or an incidental finding due to a general susceptibility toward autoimmune thyroid disease in patients with rheumatoid arthritis (8). Also, autoimmune thyroid disease has been previously reported to fluctuate between hypo- and hyperthyroidism according to the prevailing subtype of (stimulating or blocking) thyrotropin receptor antibodies (26). These were not measured in the studies included in this review. Based on the results in the present review, alterations in thyroid autoimmune status upon anti-TNF-α treatment seem to be a minor concern and may very well be overshadowed by the potential benefit of such treatment—both in regard to the rheumatoid disease and possibly the thyroid autoimmune status.

Use of RIX, a monoclonal CD20 antibody causing B-cell-depletion, did not lead to significant changes in thyroid status or autoimmunity (TPOAbs/TgAbs) in the study by Kaklamanos et al. (20). In the case by Raterman et al. (25), treatment with RIX for rheumatoid arthritis may have affected the coexisting autoimmune hypothyroidism causing a shift to a (iatrogenic) hyperthyroid state after a few months of treatment with RIX and unchanged l-thyroxine dose. However, thyrotropin receptor antibody levels were not reported in the studies and no distinction was made between thyroid disease entities (Hashimoto’s vs. Graves’ disease). In Graves’ disease complicated by moderate/severe orbitopathy, treatment with RIX has shown promising results (27, 28).

Unlike the known thyroidal side effects of immunomodulatory agents used in hepatitis C and cancer treatment, the immunomodulatory agents (anti-TNF-α and RIX) used in treatment of rheumatoid arthritis did not lead to significant changes in thyroid function nor autoimmunity. However, the well-established association between rheumatic diseases and thyroid autoimmunity necessitates continued monitoring of thyroid function in patients with rheumatoid arthritis and *vice versa* (5, 8, 29). Finally, it is advised to scrutinize each new immunomodulatory agent for its effect on thyroid as well as other autoimmune diseases and for each disease to be treated, in order to establish concise recommendations for follow-up of each agent and each disease (30).

## Author Contributions

SB and SWB are shared first authors and equally made primary contributions to data collection and analysis, interpretation of results, and writing of the manuscript. All authors contributed substantially to the study conception and design, interpretation of results, critical and intellectual revision of the manuscript, and all approved the final manuscript for publication.

## Conflict of Interest Statement

The authors declare that the research was conducted in the absence of any commercial or financial relationships that could be construed as a potential conflict of interest. The reviewer, SF, and handling editor declared their shared affiliation, and the handling editor states that the process nevertheless met the standards of a fair and objective review.
